# Adjuvant Chemotherapy in Node-Negative Advanced Gastric Cancer Patients

**DOI:** 10.1155/2022/2286040

**Published:** 2022-05-20

**Authors:** Jian-wei Su, Ya-ting Zeng, Shu-ai Luo, Yu-ying Sun, Chun-yu Huang

**Affiliations:** ^1^Department of Gastrointestinal Medicine, Affiliated Hospital of YouJiang Medical University for Nationalities, Baise, Guangxi 533000, China; ^2^State Key Laboratory of Oncology in South China, Guangzhou 510060, China; ^3^Collaborative Innovation Center for Cancer Medicine, Sun Yat-sen University Cancer Center, Guangzhou 510060, China; ^4^Department of Endoscopy, Sun Yat-sen University Cancer Center, Guangzhou 510060, China; ^5^Cancer Prevention Center, Sun Yat-sen University Cancer Center, Guangzhou 510060, China

## Abstract

Currently, there is still controversy on postoperative adjuvant chemotherapy for node-negative advanced gastric cancer. Herein, we sought to evaluate the role of postoperative adjuvant chemotherapy in these patients. We retrospectively analyzed the clinical and pathological characteristics of 363 node-negative advanced gastric cancer patients in our hospital from 1996 to 2007 who underwent gastrectomy and D2 lymphadenectomy. We compared the survival rate of the surgery-only group with that of the adjuvant chemotherapy treatment group. The 5-year survival rates of patients in the surgery-only group and the chemotherapy treatment group were 70.7% and 73.8%, respectively. There was no significant difference in the survival rate between patients receiving postoperative chemotherapy and patients not receiving chemotherapy (*P*=0.328). However, postoperative chemotherapy treatment significantly increased the survival rate of pT4aN0M0 patients (*P*=0.020), although it did not exert a direct effect on the survival rate in pT2N0M0 and pT3N0M0 patients (*P*=0.990 and *P*=0.895). We also summarized and analyzed the side effects and safety of postoperative adjuvant chemotherapy. The rate of chemotherapy-related adverse events was 79.9%. Although 61 (36.1%) patients had to adjust their chemotherapy dose, no patient died from side effects. In conclusion, postoperative chemotherapy treatment is safe but did not show a direct impact on the survival rate of the node-negative advanced gastric cancer patients. However, pT4aN0M0 patients can benefit from postoperative adjuvant chemotherapy after undergoing D2 radical resections.

## 1. Introduction

Gastric cancer is the fifth most common malignancy in the world and the third leading cause of cancer-related death in humans [1]. In China, gastric cancer was predicted to rank as the second most common cancer in 2015, with 0.3 million deaths and 0.4 million new cases reported [2]. Surgery is the main treatment for operable gastric cancer; however, its recurrence rates are high (approximately 40–80% in advanced cases) [3, 4]. Lymph node-negative gastric cancer patients are a special group of patients. Although these patients show a better survival rate than pathological lymph node-positive gastric cancer patients, recurrence occurred in many patients after radical surgery [5]. A recent series of reports have suggested that the depth of tumor invasion is an independent prognostic factor for lymph node-negative gastric cancer patients [6]. However, there is still controversy about the prognostic significance of other factors, including tumor size, lymphovascular invasion, retrieved lymph nodes, and patient age [7]. In East Asia, particularly in China, Japan, and South Korea, D2 gastrectomy is the standard surgical treatment for lymph node-negative gastric cancer [8–10]. D2 gastrectomy is also recommended in the European and US treatment guidelines for resectable diseases [11, 12]. However, adjuvant chemotherapy for lymph node-negative gastric cancer differs greatly between eastern and western countries.

Therefore, our study retrospectively analyzed the clinical and pathological characteristics of the 363 patients in our hospital from 1996 to 2007 who underwent gastrectomy and D2 lymphadenectomy. The patients were histologically negative for lymph node metastasis postoperatively, and the invasion depth was at least to the muscular layer (pT2-4N0M0). We therefore aimed to identify independent prognostic factors in this group of patients. We assigned these patients to a “surgery-only” control group or a “postoperative chemotherapy treatment” group and compared their survival rates to evaluate the role of postoperative adjuvant chemotherapy in this group of patients. We also summarized and analyzed the side effects and safety of postoperative adjuvant chemotherapy in this group of patients.

## 2. Materials and Methods

### 2.1. Patient Population

From Jan 1^st^ 1996 to May 31^st^ 2007, 1486 patients underwent D2 gastric resections at Sun Yat-sen University Cancer Center in Guangzhou, China. Among the 1486 patients, 363 cases were classified according to the International Union Against Cancer TNM system (version 8.0) as pT2-4N0M0. The research was approved by the Ethics Committee of Sun Yat-sen University Cancer Center, and written informed consent was obtained from each patient involved in the study. The inclusion criteria were as follows: (1) gastric adenocarcinoma identified by histopathological examination; (2) patients who underwent gastrectomy and D2 lymphadenectomy, were histologically proven to be free of lymph node metastasis postoperatively, and had the invasion depth at least to the muscular layer (pT2-4N0M0); (3) patients who had no preoperative cancer treatment, such as chemotherapy, immunotherapy, or radiotherapy; (4) patients with complete follow-up data, including length of survival and terminal status (survival, death, or lost to follow-up); (5) patients with the Eastern Cooperative Oncology Group performance status of 0 or 1. The exclusion criteria were as follows: (1) patients with incomplete clinical data making the statistical analyses difficult; (2) patients with did not undergo radical resection; (3) patients with a combined history of familial malignancy or other synchronous malignancy (such as gastrointestinal stromal tumor (GIST), esophageal cancer, or colorectal cancer); (4) patients who died in the perioperative period.

### 2.2. Postoperative Adjuvant Chemotherapy

All of the patients underwent D2 gastrectomy with R0 resection at Sun Yat-sen University Cancer Center. After surgery, the specimens were sent for routine pathological examinations. Approximately, 169 cases (46.6%) received adjuvant chemotherapy, while 194 (53.4%) cases did not. No patients received adjuvant radiotherapy. The chemotherapy regimens included the following: (1) single-regimen chemotherapy: 5-fluorouracil or an oral fluoropyrimidine, such as capecitabine or S1; (2) double-regimen chemotherapy: fluoropyrimidine combined with platinum or docetaxel; (3) triple-regimen chemotherapy: fluoropyrimidine and platinum-based combinations with docetaxel or epirubicin. Chemotherapy was initiated within 8 weeks of the operation, and only patients who completed at least 4 cycles of chemotherapy were regarded as effective medical cases; otherwise, they were classified as “did not receive adjuvant chemotherapy.”

### 2.3. Follow-up

Postoperative follow-up occurred at our outpatient department and included clinical and laboratory examinations every 3 months for the first 2 years, every 6 months during the third to fifth years, and annually for an additional 5 years or until patient death. The overall survival period, which was defined as the time from the operation to the time of the patient's death or last follow-up, was used as a measure of prognosis. The last follow-up date was May 31^st^ 2017.

### 2.4. Statistical Analysis

All statistical analyses were performed using Statistical Package for the Social Sciences software v17.0 for Windows (SPSS, Inc., Chicago, IL). Overall survival curves were calculated with the Kaplan–Meier method and were analyzed with the log-rank test. A Cox proportional hazard analysis was used for the univariate and multivariate analyses to explore the effect of clinicopathological variables on survival. Variables that were highly associated with others were excluded from the final multivariate Cox proportional hazards model. A *P* value < 0.05 was considered statistically significant.

## 3. Results

### 3.1. Patient Prognosis

Clinicopathological characteristics from a total of 363 node-negative advanced gastric cancer patients are presented in Table 1. At the final follow-up on May 31^st^ 2017, 224 (61.7%) patients were free of disease, 28 (7.7%) were alive with disease, and 111 (30.6%) were dead. Among the deaths, 102 patients died from the tumor and 9 died from other diseases. The follow-up period ranged from 2 months to 197 months (median, 77.0 months). The median survival time was 73.0 months, and the overall 1-year, 3-year, 5-year, and 10-year survival rates for the entire group of patients were 92.3%, 79.9%, 72.2%, and 67.5%, respectively.

### 3.2. Survival Estimates according to Clinicopathological Parameters

**Figure 1:**
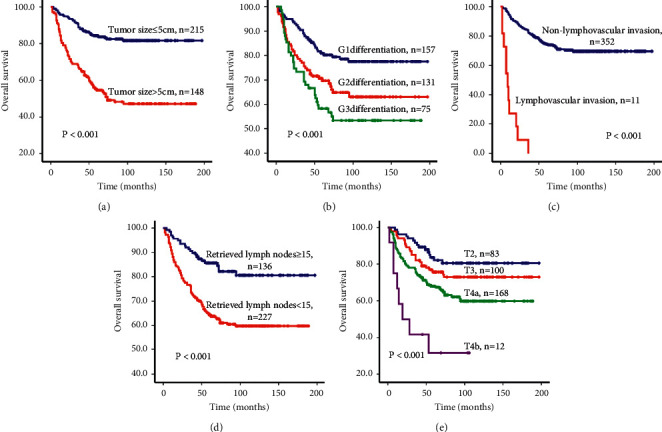
Kaplan–Meier curves and log-rank test results of survival analyses of patients with node-negative advanced gastric cancer based on the clinicopathological parameters. (a) Patients with tumor size ≤5 cm or tumor size >5 cm. (b) Patients with G1, G2, or G3 differentiation. (c) Patients with non-lymphovascular invasion or lymphovascular invasion. (d) Patients with retrieved lymph nodes ≥15 or retrieved lymph nodes <15. (e) Patients with T2, T3, T4a, or T4b stage.

The Kaplan–Meier method and log-rank test were performed to evaluate the prognostic value of patients' clinicopathological parameters. The results showed that patients with tumor size ≤5 cm, G1 differentiation, nonlymphovascular invasion, retrieved lymph nodes ≥15, and T2/T3 stage had a significantly better survival rate than those with tumor size >5 cm, G2/G3 differentiation, lymphovascular invasion, retrieved lymph nodes <15, and T4 stage (Figure 1).

The effects of the clinicopathological parameters on the prognosis of postgastrectomy node-negative advanced gastric cancer patients were further evaluated by univariate and multivariate Cox proportional hazards regression analyses. Based on a univariate analysis that included all 363 patients, tumor size, differentiation, lymphovascular invasion, retrieved lymph nodes, and the T stage were also found to have statistically significant associations with overall survival (Table 2). All these factors were included in a multivariate Cox proportional hazards model to adjust for the effects of the covariates. Based on this model, tumor size, differentiation, lymphovascular invasion, retrieved lymph nodes, and the T stage remained independent prognostic factors (Table 2).

### 3.3. Relationship between Postoperative Adjuvant Chemotherapy and Survival

**Figure 2:**
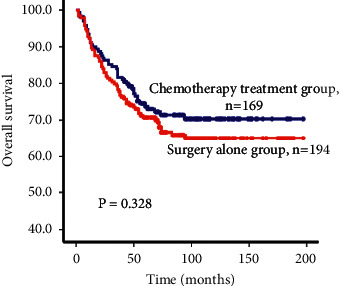
Kaplan–Meier survival curves for node-negative advanced gastric cancer patients who received postoperative chemotherapy treatment (*n* = 169) or surgery-alone therapy (*n* = 194). The log-rank test showed that there was no significant difference in survival rate between the two groups.

The clinicopathological parameters between the surgery-only control group and postoperative chemotherapy treatment group were well matched. There were no statistically significant differences between the two groups for all variables that were tested (*P* ≥ 0.05, Table 1). The patients who received postoperative chemotherapy treatment did not exhibit a better survival rate than the surgery-only control group (Figure 2). The median survival time for patients in the chemotherapy treatment group was 73 months compared with 72 months for patients in the surgery-only control group. The survival rates at 1, 3, 5, and 10 years were 93.1%, 81.7%, 73.8%, and 70.3%, respectively, for the chemotherapy treatment group compared to 91.2%, 78.4%, 70.1%, and 65.1% for the surgery-only control group (log-rank test, *P*=0.328). Univariate analyses also did not show a direct relationship between postoperative chemotherapy treatment and survival benefits in the patients (Table 2).

### 3.4. Subgroup Analysis

**Figure 3:**
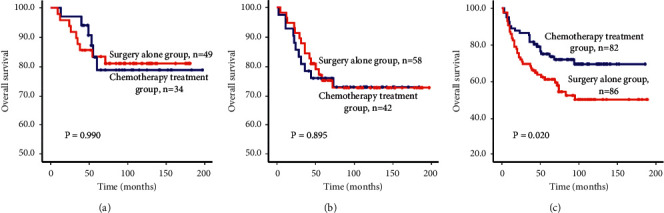
Kaplan-Meier survival curves for the patients according to T stage. (a) pT2N0M0 gastric cancer patients. (b) pT3N0M0 gastric cancer patients. (c) pT4aN0M0 gastric cancer patients.

There were 83 cases of pT2N0M0 (stage Ib) tumors in this group, and 34 (41.0%) of those received postoperative adjuvant chemotherapy; the remaining 49 (59.0%) cases did not receive chemotherapy.  Figure 3(a) shows the survival rates of pT2N0M0 patients in the surgery-only group and the chemotherapy treatment group: 95.9% versus 97.1% (1 year), 87.8% versus 94.1% (3 years), 83.5% versus 78.9% (5 years), and 81.1% versus 78.9% (10 years), respectively. The survival curve of the patients receiving postoperative chemotherapy was not significantly different from that of patients who did not receive chemotherapy (*P*=0.990).

There were 100 patients with pT3N0M0 (stage IIa) tumors, and 42 (42.0%) received postoperative adjuvant chemotherapy; the remaining 58 (58.0%) cases did not receive therapy.  Figure 3(b) shows the survival rates of pT3N0M0 patients in the surgery-only group and the chemotherapy treatment group: 96.6% versus 92.9% (1 year), 84.5% versus 78.6% (3 years), 75.3% versus 76.1% (5 years), and 72.9% versus 73.1% (10 years), respectively. The survival curve of the patients receiving postoperative chemotherapy was not significantly different from that of patients who did not receive chemotherapy (*P*=0.895).

There were 168 cases of pT4aN0M0 (stage IIb) cancer, and 82 (48.8%) cases received postoperative adjuvant chemotherapy.  Figure 3(c) shows the survival rates of pT4aN0M0 patients in the surgery-only group and the chemotherapy treatment group: 86.0% versus 89.0% (1 year), 69.8% versus 85.4% (3 years), 61.2% versus 75.1% (5 years), and 50.3% versus 69.6% (10 years), respectively. The survival curve of the patients receiving postoperative chemotherapy significantly improved compared to those who did not receive chemotherapy (*P*=0.020).

There were 12 cases of pT4bN0M0 (stage IIIb) cancer, and 11 received postoperative chemotherapy. There were too few cases to conduct a survival analysis.

### 3.5. Chemotherapy-Related Adverse Events

The rate of chemotherapy-related adverse events was 79.9%, and the most common toxicities were digestive system toxicity, hematological toxicity, and peripheral nerve toxicity. The third-fourth-degree toxicities primarily included digestive system toxicities and hematological toxicities: nausea (7.7%), vomiting (5.3%), reduction of neutrophil granulocytes (18.9%), and platelet reduction (7.1%). Furthermore, 3 patients had third-fourth-degree peripheral neuropathy. No deaths were related to chemotherapy (Table 3).

Due to adverse events, 61 (36.1%) patients had to adjust their chemotherapy dose. The most common reasons were neutrophil reduction, nausea, vomiting, platelet reduction, and anorexia. 11 (6.5%) patients had treatment interruption due to adverse events, which mainly included neutrophil reduction (*n* = 4; 2.4%), platelet reduction (*n* = 2; 1.2%), vomiting (*n* = 3; 1.8%), and severe peripheral neuropathy (*n* = 2; 1.2%).

## 4. Discussion

The famous MAGIC study established perioperative chemotherapy as an effective regimen for resectable gastric cancer [13]. Patients who received the ECF regimen (or its modifications) as new adjuvant chemotherapy are recommended to follow the MAGIC research procedures and receive more than three cycles of ECF or its modifications. This has already obtained expert consensus with category I evidence [12]. However, the western and eastern guidelines differ on the use of adjuvant chemotherapy in patients who did not receive ECF or its modifications before surgical resection. The Intergroup-0116 (INT-0116) study [14] demonstrated the effectiveness of postoperative chemoradiotherapy in treating adenocarcinoma of the gastric or gastroesophageal junction. The relapse-free survival rate (48% vs. 31%) and the overall survival rate (50% vs. 41%) improved (*P*=0.005). However, this study was controversial (particularly in Asia) because more than 90% of the included cases underwent D0/D1 surgery. Some gastric cancer experts in Asia agreed that more studies were required to demonstrate the role of adjuvant chemoradiotherapy in patients who undergo D2 surgery.

In 2007, the Japanese reported the results of the ACTS-GC study (JCOG9912) [15], and S1 demonstrated success in gastric cancer adjuvant chemotherapy. In Japan, based on the Adjuvant Chemotherapy Trial, TS-1 is recommended for stage II and IIIa gastric cancer patients after D2 gastrectomy. However, these results could not be successfully repeated in Europe and America and are therefore not acknowledged in western countries. In 2012, the CLASSIC study [16] published in *Lancet* showed that a 6-month course of chemotherapy (capecitabine and oxaliplatin) after D2 gastrectomy improved 3-year disease-free survival rate compared to surgery alone. Chemotherapy reduced the relative risk of disease recurrence, new disease occurrence, and death compared to surgery alone. Moreover, a subgroup analysis suggested that adjuvant chemotherapy was beneficial for all disease stages (II, IIIa, or IIIb). Unfortunately, the research subjects were limited to South Koreans and Chinese, and so there was a lack of data in western populations. More clinical evidence is required to demonstrate the value of adjuvant chemotherapy for gastric cancer after D2 surgery.

Lymph node-negative gastric cancer patients are a special group of patients. Although, D2 surgery should be performed in this group of patients, the treatment with postoperative adjuvant chemotherapy for these patients is still greatly different between eastern and western countries. In order to get the best treatment for lymph node-negative gastric cancer, our research retrospectively analyzed the patients' clinical and pathological data and compared the survival rate of the surgery-only group and the adjuvant chemotherapy treatment group. From Jan 1^st^ 1996 to May 31^st^ 2007, there were 363 gastric cancer patients with pT2-4N0M0 tumors who underwent D2 surgery at our center. Among them, 169 patients received postoperative adjuvant chemotherapy. The rate of total chemotherapy-related adverse events was 79.9%. Although 61 (36.1%) patients had to adjust their chemotherapy dose, no deaths were related to chemotherapy. The toxicity is moderate, and it is safe to administer postoperative adjuvant chemotherapy for this group of patients. However, our study indicated that neither the pT2N0M0 nor the pT3N0M0 patients benefited from postoperative adjuvant chemotherapy after undergoing D2 radical resections. Because most Chinese gastric cancer patients are still rather poor, to reduce the economic and social burden, the decision of postoperative chemotherapy for patients with pT2N0M0 and pT3N0M0 tumors should be cautious and considerate. However, pT4aN0M0 patients can benefit from adjuvant chemotherapy, and based on this result, postoperative adjuvant chemotherapy is recommended for gastric cancer patients with pT4aN0M0 tumors after undergoing D2 surgery.

Meanwhile, in our study, all of the patients underwent gastrectomy and D2 lymphadenectomy, and based on our center's data, their survival was much better than those who received only palliative operations [17]. In addition, if the number of dissected lymph nodes in lymph node-negative gastric cancer patients was greater than 15, the prognosis was better than those with less than 15 dissected nodes. Morgan et al. [18], Giuliani A et al. [19], and Zhao et al. [20] agreed with the opinion that the number of dissected lymph nodes should be more than 15. Our study demonstrates that postoperative chemotherapy treatment did not show a direct impact on the survival of this group of patients. Therefore, even in node-negative advanced gastric cancer patients, D2 surgery should be performed and a sufficient number of lymph nodes should be dissected.

In conclusion, D2 surgery should be performed in node-negative advanced gastric cancer patients and the number of dissected lymph nodes should be more than 15. In addition, postoperative adjuvant chemotherapy should be administered in accordance with the tumor stage and the patient's condition. Because this was a retrospective analysis and not a prospective randomized clinical trial and there were differences in the adjuvant chemotherapy drugs, doses, and intensities administered, there may be some bias. Therefore, the results of this study are only preliminary results. More clinical trials are required to prove the value of adjuvant chemotherapy for advanced gastric cancer patients.

## Figures and Tables

**Table 1:** Demographic and clinical characteristics: surgery-only patients and adjuvant chemotherapy treatment patients.

Variables	Number	Surgery group	Chemotherapy group	*P* value
All cases	363	194	169	
Age (years)				0.373
≤60	206	114	92	
>60	157	80	77	
Gender				0.295
Male	250	129	121	
Female	113	65	48	
Location				0.589
Distal	143	73	70	
Proximal	211	115	96	
Total	9	6	3	
Tumor size(cm)				0.796
<5	215	114	101	
≥5	148	80	68	
Differentiation				0.102
G1	157	82	75	
G3/G2	206	112	94	
T stage				0.675
T2	83	47	37	
T3	100	55	45	
T4a	168	86	82	
Tb	12	6	5	
Retrieved lymph nodes				0.423
<15	227	125	102	
≥15	136	69	67	
Lymphovascular invasion				0.193
No	352	186	166	
Yes	11	8	3	

**Table 2:** Univariate and multivariate survival analysis of clinicopathologic variables in 363 cases of gastric carcinoma patients.

Variables	Univariate analysis			Multivariate analysis		
	HR	95% CI	*P* value	HR	95% CI	*P* value
Gender (male vs. female)	0.846	0.555–1.292	0.439			
Age (year) (≥60 vs. <60)	1.313	0.896–1.922	0.162			
Location (distal/proximal/total)	1.047	0.737–1.487	0.799			
Size (cm) (<2.5/2.5–5/>5)	2.980	2.115–4.199	<0.001^*∗*^	3.105	2.038–4.730	<0.001^*∗*^
Differentiation (G3/G2 vs. G1)	1.633	1.286–2.074	<0.001^*∗*^	1.553	1.221–1.974	<0.001^*∗*^
Lymphovascular invasion (no vs. yes)	16.380	8.441–31.787	<0.001^*∗*^	8.540	4.285–17.021	<0.001^*∗*^
Chemotherapy (yes vs. no)	1.206	0.827–1.757	0.333			
T stage (T4b/T4a/T3/T2)	1.717	1.335–2.207	<0.001^*∗*^	1.332	1.024–1.734	0.033^*∗*^
Retrieved lymph nodes (<15 vs. ≥15)	0.347	0.213–0.565	<0.001^*∗*^	0.414	0.253–0.678	0.001^*∗*^

HR, hazard ratio; CI, confidence interval; AFP, alpha-fetoprotein. ^*∗*^Statistically significant (*P* < 0.05).

**Table 3:** Adverse events of the chemotherapy group (*n* = 169).

	All grades	Grade 3 or 4
Patients with ≥1 adverse event	135 (79.9%)	65 (38.5%)
Nausea	110 (65.1%)	13 (7.7%)
Neutropenia	101 (59.8%)	32 (18.9%)
Decreased appetite	100 (59.2%)	9 (5.3%)
Peripheral neuropathy	25 (14.8%)	3 (1.8%)
Diarrhoea	32 (18.9%)	4 (2.4%)
Vomiting	81 (47.9%)	9 (5.3%)
Fatigue	52 (30.8%)	8 (4.7%)
Thrombocytopenia	31 (18.3%)	12 (7.1%)
Hand-foot syndrome	20 (11.8%)	2 (1.2%)
Abdominal pain	23 (13.6%)	3 (1.8%)
Stomatitis	18 (10.7%)	1 (0.6%)

## Data Availability

No data were used to support this study.

## Additional Points

As a result of these findings, D2 gastrectomy is now recommended in the European and US treatment guidelines for resectable disease.
